# Real-time computer-aided diagnosis of focal pancreatic masses from endoscopic ultrasound imaging based on a hybrid convolutional and long short-term memory neural network model

**DOI:** 10.1371/journal.pone.0251701

**Published:** 2021-06-28

**Authors:** Anca Loredana Udriștoiu, Irina Mihaela Cazacu, Lucian Gheorghe Gruionu, Gabriel Gruionu, Andreea Valentina Iacob, Daniela Elena Burtea, Bogdan Silviu Ungureanu, Mădălin Ionuț Costache, Alina Constantin, Carmen Florina Popescu, Ștefan Udriștoiu, Adrian Săftoiu

**Affiliations:** 1 Faculty of Automation, Computers and Electronics, University of Craiova, Craiova, Romania; 2 Research Center of Gastroenterology and Hepatology Craiova, University of Medicine and Pharmacy Craiova, Craiova, Romania; 3 Faculty of Mechanics, University of Craiova, Craiova, Romania; 4 Department of Medicine, Indiana University School of Medicine, Indianapolis, Indiana, United States of America; 5 Gastroenterology Department, Ponderas Academic Hospital, Bucharest, Romania; 6 Pathology Department, Emergency County Clinical Hospital Craiova, Craiova, Romania; 7 INNES Worldwide LLC, Craiova, Romania; University of Nebraska Medical Center, UNITED STATES

## Abstract

Differential diagnosis of focal pancreatic masses is based on endoscopic ultrasound (EUS) guided fine needle aspiration biopsy (EUS-FNA/FNB). Several imaging techniques (i.e. gray-scale, color Doppler, contrast-enhancement and elastography) are used for differential diagnosis. However, diagnosis remains highly operator dependent. To address this problem, machine learning algorithms (MLA) can generate an automatic computer-aided diagnosis (CAD) by analyzing a large number of clinical images in real-time. We aimed to develop a MLA to characterize focal pancreatic masses during the EUS procedure. The study included 65 patients with focal pancreatic masses, with 20 EUS images selected from each patient (grayscale, color Doppler, arterial and venous phase contrast-enhancement and elastography). Images were classified based on cytopathology exam as: chronic pseudotumoral pancreatitis (CPP), neuroendocrine tumor (PNET) and ductal adenocarcinoma (PDAC). The MLA is based on a deep learning method which combines convolutional (CNN) and long short-term memory (LSTM) neural networks. 2688 images were used for training and 672 images for testing the deep learning models. The CNN was developed to identify the discriminative features of images, while a LSTM neural network was used to extract the dependencies between images. The model predicted the clinical diagnosis with an area under curve index of 0.98 and an overall accuracy of 98.26%. The negative (NPV) and positive (PPV) predictive values and the corresponding 95% confidential intervals (CI) are 96.7%, [94.5, 98.9] and 98.1%, [96.81, 99.4] for PDAC, 96.5%, [94.1, 98.8], and 99.7%, [99.3, 100] for CPP, and 98.9%, [97.5, 100] and 98.3%, [97.1, 99.4] for PNET. Following further validation on a independent test cohort, this method could become an efficient CAD tool to differentiate focal pancreatic masses in real-time.

## Introduction

The diagnosis of pancreatic cancer has a grim prognosis, with a 5-year survival rate less than 10%, so there is an urgent need for better early detection and treatment options [1]. Pancreatic cancer incidence and mortality rates have increased significantly over the last decades [2], in part because pancreatic cancer is difficult to diagnose until the disease has reached an advanced stage. Its accurate diagnosis relies on modern imaging such as endoscopic ultrasound (EUS), endoscopic retrograde cholangiopancreaticography (ERCP) or multi-detector CT angiography performed using a dual-phase pancreatic protocol. Accumulated evidence has revealed that EUS, contrast-enhanced EUS (CE-EUS) and EUS elastography play an important role the differential diagnosis of pancreatic solid lesions and clinical evaluation of pancreatic cancer [3].

The important improvements of deep learning and other machine learning techniques are expected to produce a big impact in medical images diagnosis, however these techniques are currently underdeveloped [4]. Among the machine learning algorithms related to image feature extraction and classification, CNNs have been widely proven to be superior to traditional algorithms. These networks provide the flexibility of extracting discriminative features from images preserving the spatial structure and could be developed for region recognition and classification of medical images. Many studies on abdominal imaging were conducted to localize and segment organs such as liver, kidneys, bladder, and pancreas [5–7]. The imaging modality used was either MRI for prostate analysis or CT for other organs [4]. In these previous studies, the combination between CNN and LSTM was used to discover the time dependencies in images sequences for falling detection [8].

In the current study, we used two deep learning techniques, the Convolution Neural Network (CNN) and Long Short-term Memory (LSTM) models to detect the focal pancreatic masses in four EUS imaging modalities (gray-scale, color Doppler, contrast-enhancement and elastography).

## Material and methods

### Patient data

Data from 65 patients with focal pancreatic masses were included in the study, with a total of 20 images selected for each patient from the movies stored on the embedded HDD of the ultrasound system, 5 images each of EUS: gray-scale (B-mode), low-MI contrast-enhancement (CHI) (arterial and venous phase), high-MI color Doppler (CDI), real-time elastography (RTE). The study protocol was approved by the Research and Ethics Committee of the University of Medicine and Pharmacy of Craiova and carried out in accordance with the Code of Ethics of the World Medical Association (Declaration of Helsinki) for experiments involving humans. All patients received and signed a written informed consent.

The final diagnosis has been confirmed through cytopathological analysis of EUS-FNA/FNB samples, with a follow-up of over 12 months for the patients and categorized as follows: chronic pseudotumoral pancreatitis (CPP), pancreatic neuroendocrine tumor (PNET) and pancreatic ductal adenocarcinoma (PDAC).

#### Multiparametric EUS

The imaging equipment consisted of a linear EUS (EG 3870 UTK, Pentax Medical Corporation) coupled with a high-end ultrasound system (Hitachi Preirus). Each lesion was assessed using:

- Gray-scale (B-mode);
- Contrast-enhancement (Sonovue/Lumason 4.8 mL): pre-contrast (low-MI), post-contrast (10s and 20s) for the arterial phase, and post-contrast (30s and 40s) for the venous phase;
- Color Doppler, post-contrast (high-MI);
- Real-time elastography.

#### Disease patterns

**Fig 1:**
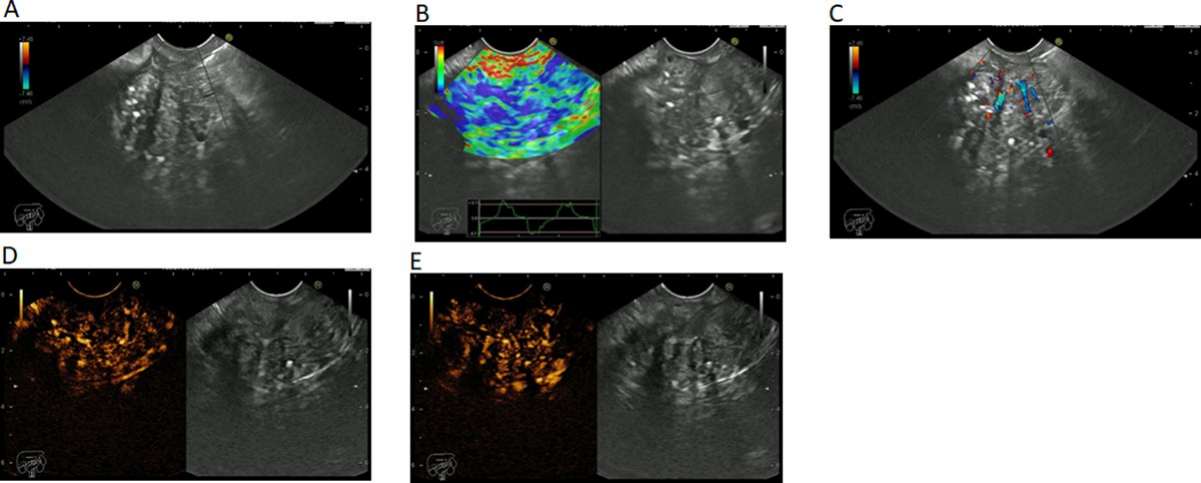
EUS imaging of a pseudotumoral chronic pancreatitis in (A) gray scale. (B) elastography. (C) color Doppler. (D) contrast enhancement–arterial phase. (E) contrast enhancement–venous phase.

EUS images of CPP were characterized by hypoechoic masses in gray-scale; hyper-enhanced with low-MI CHI in arterial phase and venous phase; hyper-enhanced with CDI and intermediate stiffness with RTE (Fig 1).

**Fig 2:**
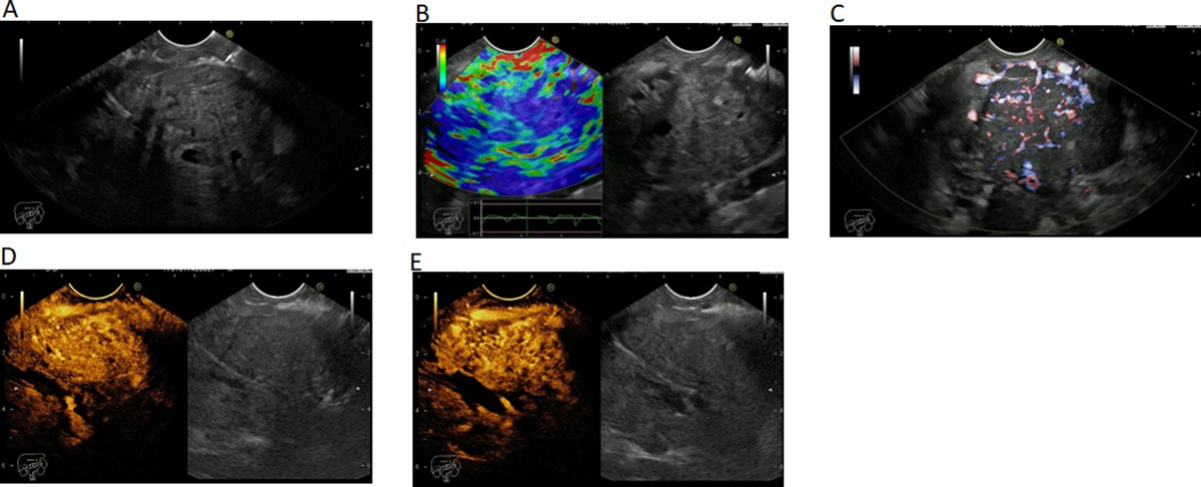
EUS imaging of a neuroendocrine tumor in (A) gray scale. (B) elastography. (C) color Doppler. (D) contrast enhancement–arterial phase. (E) contrast enhancement–venous phase.

The PNET masses had the following features on EUS: hypoechoic mass in gray-scale; hyper-enhanced with low-MI CHI in arterial phase and venous phase (wash-out); hyper-enhanced with high-MI CDI and high stiffness with RTE (Fig 2).

**Fig 3:**
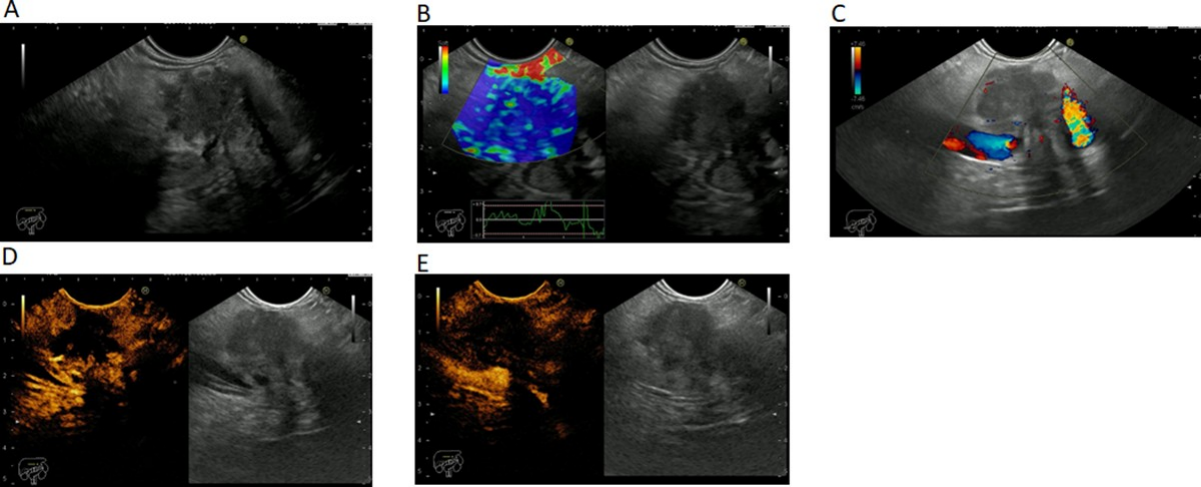
EUS imaging of a pancreatic ductal adenocarcinoma in (A) gray scale. (B) elastography. (C) color Doppler. (D) contrast enhancement–arterial phase. (E) contrast enhancement–venous phase.

PDAC images were characterized by the following patterns: hypoechoic mass in gray-scale; hypo-enhanced with low-MI CHI in the arterial and venous phase; hypo-enhanced with high-MI CDI and high stiffness with RTE (Fig 3).

### CNN and LSTM model development

We developed a pancreatic diagnosis prediction method which combines CNN with LSTM to automatically analyze the sequential and multistate pancreatic images. A CNN-LSTM was designed for sequence diagnosis problems with spatial inputs, like pancreatic images and videos. Our CNN-LSTM model could effectively encode spatio-temporal information and extract high-level representations. CNN assume that all inputs and outputs are independent of each other, while the basic assumption of LSTM is that there is an interaction between the input sequences [9]. Subsequently, the extracted dependencies of the data features were used to improve the recognition accuracy. Each image modality was integrated through its dedicated module and the extracted descriptors were then concatenated to perform the final classification. The integration of information from the four modalities (gray-scale, color Doppler, contrast-enhancement and elastography) ensured that complementary features for diagnosis learning are extracted. The model was developed in three steps:

1. We used the CNN model to extract the spatial features of images from four types of pancreatic imaging modalities: *gray scale*, *contrast harmonic sequential images* taken at 0, 10, 20, 30, 40 seconds, *color Doppler*, and respectively *real-time elastography imaging*.
2. We applied the LSTM network to extract the temporal information of the sequential images of the contrast harmonic imaging taken at 0, 10, 20, 30, 40 seconds.
3. We used a concatenation layer to integrate the feature vectors. After merging the features, a fully connected layer (FC3) and a *softmax* function were used for the pancreatic diagnosis prediction.

For each type of imaging modality, we have developed a CNN with 4 convolutional layers with a feature map of size (3 x 3), 3 max-pooling layers with a pooling window of size (2 x 2), 2 dropout layers and 2 fully connected (dense) layers. The final dense layer has 3 outputs and a *softmax* activation. The *convolutional layers* filter the images by detecting patterns at different locations in the image. A *pooling layers* follows a convolution layer to down-sample the features from the previous convolution layer, such that every feature map contained in a pooling layer is connected with a feature map in the convolution layer. LSTM network models are a type of recurrent neural network that are able to learn and remember over long sequences of input data; it detects and locates patterns inside images sequences and extracts the temporal information [9].

**Fig 4:**
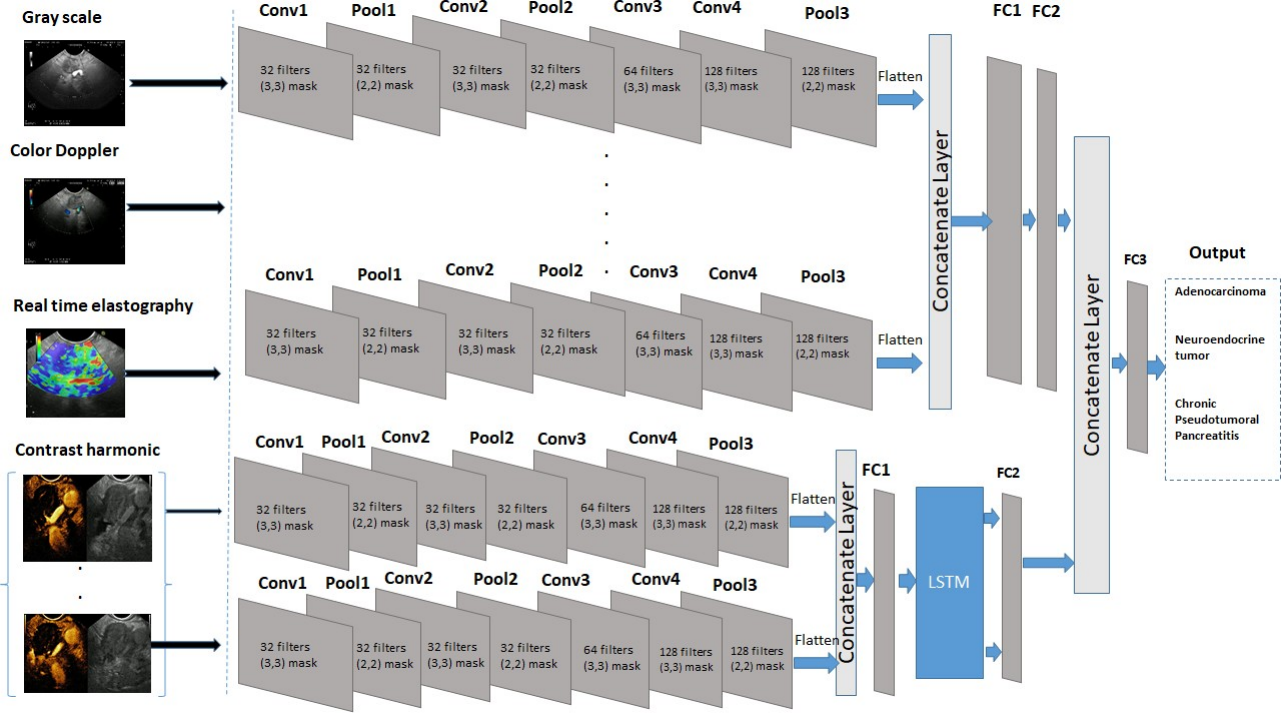
The architecture of the CNN-LSTM model.

The parameters of the model are listed in S1 Table (see S1 File). The model architecture is shown in Fig 4. A detailed architecture of the proposed CNN_LSTM model is shown in S1 Fig (see S1 File).

#### Model regularization

We have applied different techniques in order to reduce the overfitting. Rectified linear unit [10] was applied for non-linear activation function. The dropout method [11] was used to randomly deactivate a fraction of the units or connections in the network on each training iteration in order to help the network to be capable of better generalization and to avoid overfitting of training data. The data augmentation technique generated more training data from existing images by augmenting the samples via a number of random transformations. The goal was to obtain different images for training our model so that it did not use the same image twice.

#### Image dataset set-up

The initial set of 1300 images was augmented as described below resulting in a final dataset of 3360 images. Of the total set of 3360 focal pancreatic masses images, 2688 images were used for training and 672 images for testing (Table 1).

**Table 1:** The distribution of the images and patients for training/validation and testing datasets.

Image Datasets	PDAC (class 0) Images/Patients	CPP (class 1) Images/Patients	PNET (class 2) Images/Patients	Total Images/Patients
**Training/ Validation**	992/30	896/20	800/15	2688/65
**Testing**	248/30	224/20	200/15	672/65
**Total**	1240/30	1120/20	1000/15	3360/65

PDAC: pancreatic ductal adenocarcinoma; CPP: chronic pseudotumoral pancreatitis; PNET: pancreatic neuroendocrine tumor.

The initial large size of the pancreatic mass images (1024 x 768px) contained the clinical region of interest in the middle and adjacent tissue on every side. Therefore, by selecting up to four 500x500px sub-areas from the original image which contained the area of interest and some of the adjacent tissue, we could increase the image data set while maintaining relevant clinical data in each sub-areas. More precisely, the transformations we applied to the image dataset were:

- Automatic cropping of the image border which contained information about equipment and patient’s identification.
- Manual selection of either one (PDAC) or between 2–4 (PNET and CPP diagnosis classes) 500x500px images from each initial one of the 1300 1024x768px original images. For the PNET and CPP diagnosis classes, the 500x500px images were selected such that each included the region of interest and a variable area of adjacent tissue on the right, top or bottom of the region of interest.
- The final dataset of 3360 500x500px images was obtained by performing several additional automatic transformations which are practiced routinely in CNN studies: width_shift and height_shift to randomly translate pictures vertically or horizontally, shear_range to randomly apply shearing transformations, and zoom_range to randomly zoom in images [12].

The CNN datasets were developed using the train-validation-test pattern (Table 1). The validation and testing datasets are the same, but different from the training dataset. For each patient we randomly selected four images from each imaging modality for training dataset and one for the testing dataset. Therefore 80% of images were chosen randomly for validation/training and 20% for testing. The testing dataset was used to monitor progress during epochs, and possibly early stopping but not for gradient descent.

The network was trained using the RMSProp optimization algorithm [12] on a single NVIDIA Quadro K4200 GPU for 50 epochs. Intel(R) Xeon(R) CPU E5-1620 v3 @ 3.50GHz, 32 GB RAM architecture was used to run the experiments. Keras with Google TensorFlow backend was used to implement the CNN and LSTM model in this study, together with other scientific computing libraries as numpy and scikit-learn [12–14].

#### Evaluation metrics

To analyze the performance of our CNN-LSTM method, we used the following medical diagnosis metrics: specificity (Sp) (1), sensitivity (Se) (2), negative predictive value (NPV) (3), positive predictive value (PPV) (4), the test accuracy (5), the area under curve (AUC) and precision recall curves. In addition, we computed the 95% confidence intervals (CI) for NPV and PPV [15].


$$Sp=\frac{\left|true\;negative\right|}{\left|true\;negative|+|false\;positive\right|}$$ (1)

$$Se=\frac{\left|true\;positive\right|}{\left|true\;positive|+|false\;negative\right|}$$ (2)

$$NPV=\frac{\left|true\;negative\right|}{\left|true\;negative|+|false\;negative\right|}$$ (3)

$$PPV=\frac{\left|true\;positive\right|}{\left|true\;positive|+|false\;positive\right|}$$ (4)

$$Accuracy=\frac{\left|correctly\;classified\;cases\right|}{\left|test\;cases\right|}$$ (5)

Another important diagnostic tool we used for the analysis of probabilistic prediction of multi-class classification was the receiver operating characteristic (ROC) curves. The ROC Curves summarize the trade-off between the true positive rate and false positive rate for a predictive model using different probability thresholds. This means that the top left corner of the plot is the ideal point with a false positive rate of zero, and a true positive rate of one. We computed the micro and macro averaging to evaluate the overall performance across all classes. In micro averaging, we computed the performance from the individual true positives, true negatives, false positives, and false negatives of each diagnosis class. In macro averaging we computed the average performance of each diagnosis classes.

We used the Precision-Recall as another metrics of the model’s prediction [16]. Precision is a measure of the ability of the CNN-LSTM model to identify only the relevant diagnosis, while recall is a measure of the ability of the model to find all the relevant cases within the dataset.

## Results and discussion

**Fig 5:**
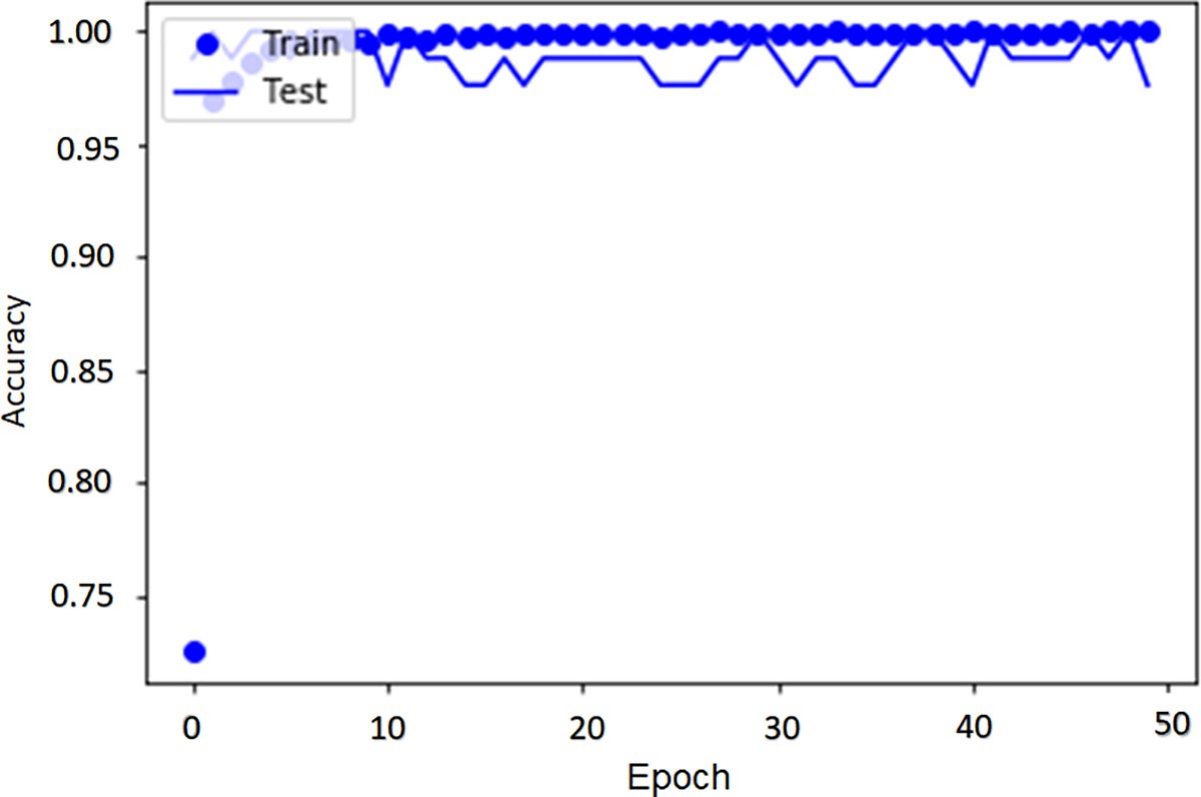
The comparison between the accuracy of the training and the testing datasets.

**Fig 6:**
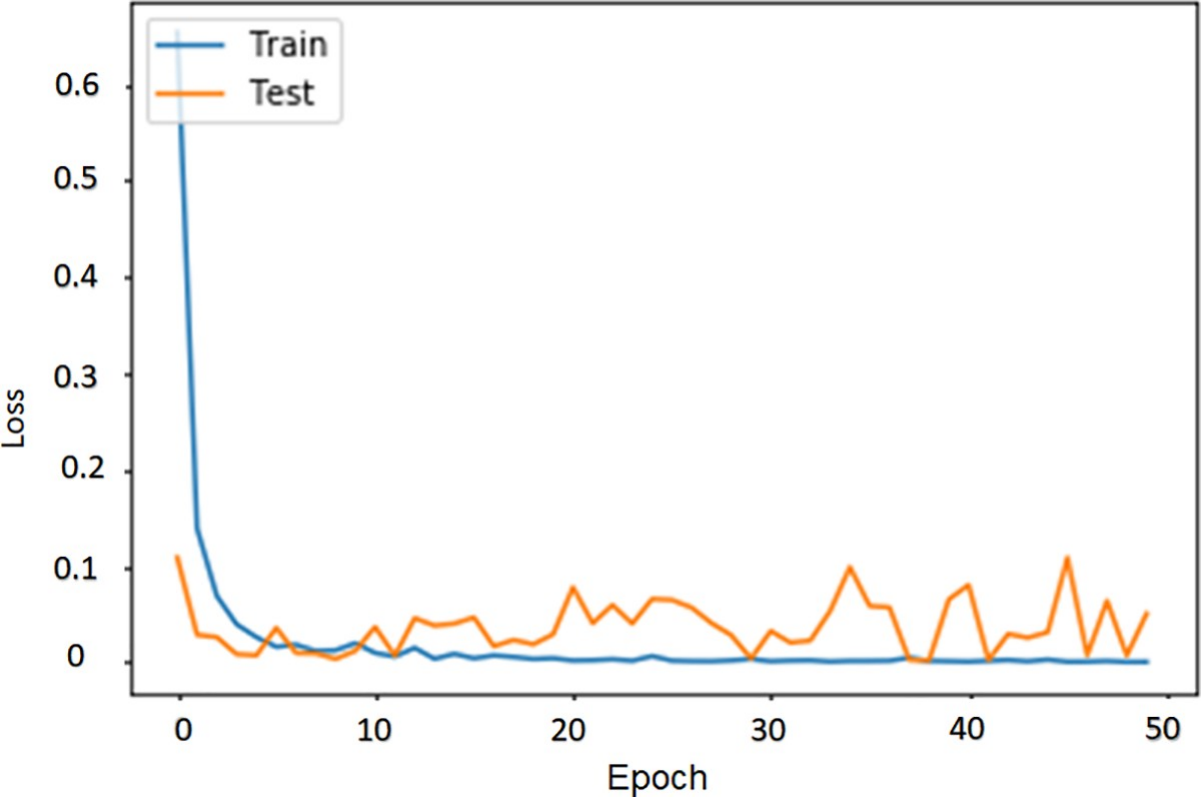
The comparison between the loss of the training and validation datasets.

The algorithm was trained for 50 epochs (iterations over the whole training and testing datasets). The results of the convolution operation are obtained from the 128x128px versions of the 500x500px images. This is an established method for balancing the algorithm’s ability to analyze relevant anatomical features in every image, and the computational burden (GPU memory and running speed) [17]. The algorithm returns the loss and accuracy on the training and testing datasets for each epoch and a final classification error rate and accuracy. The final test accuracy was 98.26%. The training dataset curves closely tracked the testing dataset curves (Figs 5 and 6).

The overall sensitivity of the model for the diagnosis of focal pancreatic masses was 98.60%%, with a specificity of 97.40%. As shown in Table 2, a balance exists between sensitivity and specificity which is extremely important in diagnosis decision. The CNN-LSTM model achieved an overall accuracy of 97.61% for the diagnosis of PDAC, with a sensitivity of 98.11% (with [0.968,0.994] 95% CI) and a specificity of 96.77% (with [0.945,0.989] 95% CI). The NPV for the PDAC diagnosis was 96.7% and the corresponding 95%CI was [94.5%, 98.9%]. The PPV of the PDAC diagnosis was 98.1% and the corresponding 95% CI was [96.8%, 99.4%]. All metrics computed for CNN-LSTM model and the corresponding 95% CIs are summarized in Table 2.

**Table 2:** Evaluation metrics for each diagnosis class.

Diagnosis	Accuracy 95% CI (%)	AUC 95% CI (%)	Sp 95% CI (%)	Se 95% CI (%)	NPV 95% CI (%)	PPV 95% CI (%)
**PDAC**	97.61 [96.4, 98.7]	0.97 [0.967, 0.992]	96.7 [94.5, 98.9]	98.11 [9.68, 99.4]	96.7 [94.5, 98.9]	98.1 [96.81, 99.4]
**CPP**	98.66 [97.7, 99.5]	0.99 [0.98,0.999]	99.55 [98.6, 100]	98.21 [96.9, 99.4]	96.5 [94.1, 98.8]	99.7 [99.3, 100]
**PNET**	98.51 [97.5, 99.4]	0.98 [0.966,0.993]	95.9 [93.2, 98.7]	99.5 [98.9,100]	98.9 [97.5, 100]	98.3 [97.1, 99.4]
**Mean**	98.26	0.98	97.4	98.6	97.4	98.7

CI: confidence interval; AUC: area under the curve; Sp: specificity; Se: sensitivity; NPV: negative predictive value; PPV: positive predictive value; PDAC: pancreatic ductal adenocarcinoma; CPP: chronic pseudotumoral pancreatitis; PNET: pancreatic neuroendocrine tumor.

**Fig 7:**
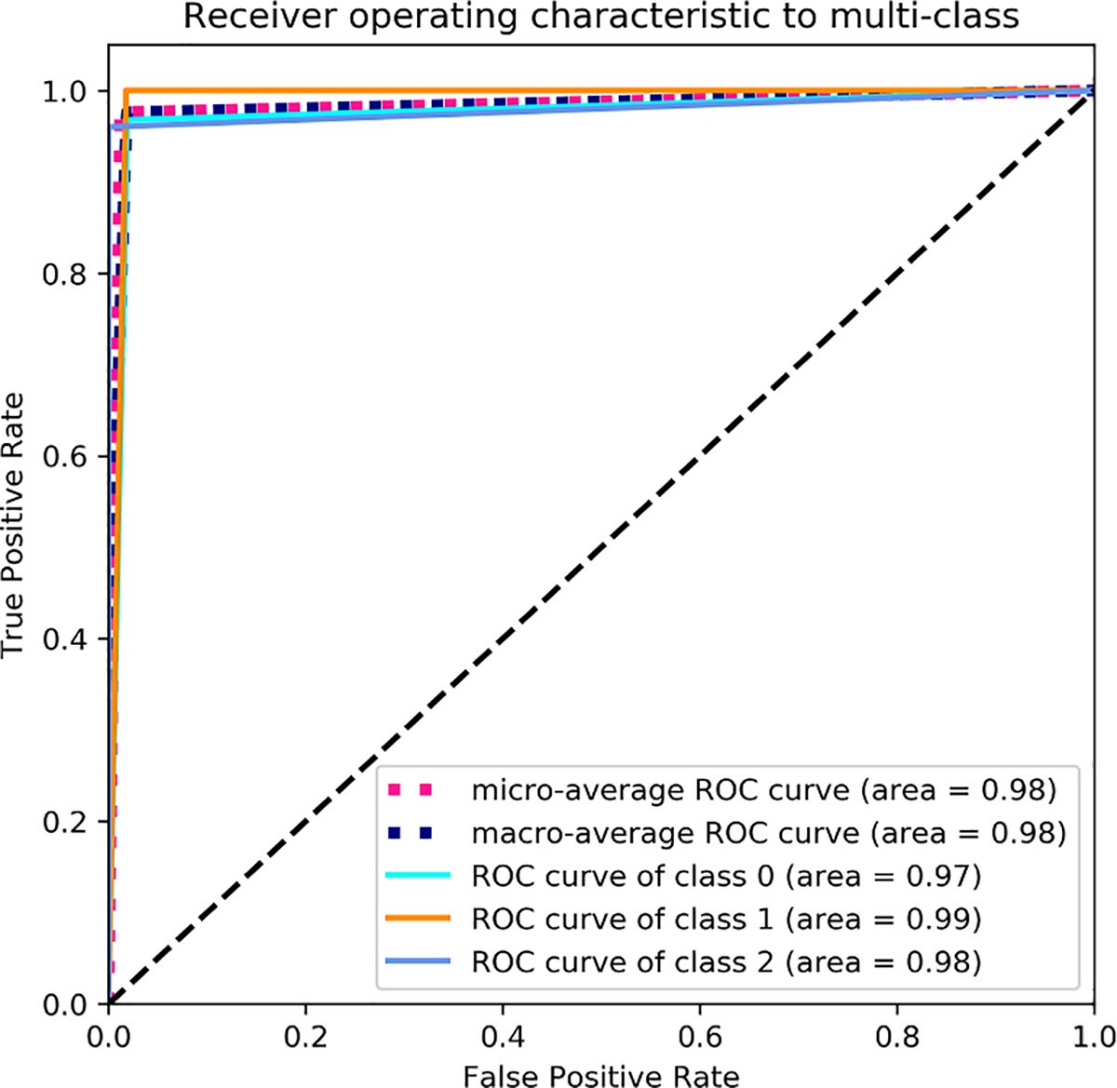
The ROC curves computed for CNN-LSTM method.

**Fig 8:**
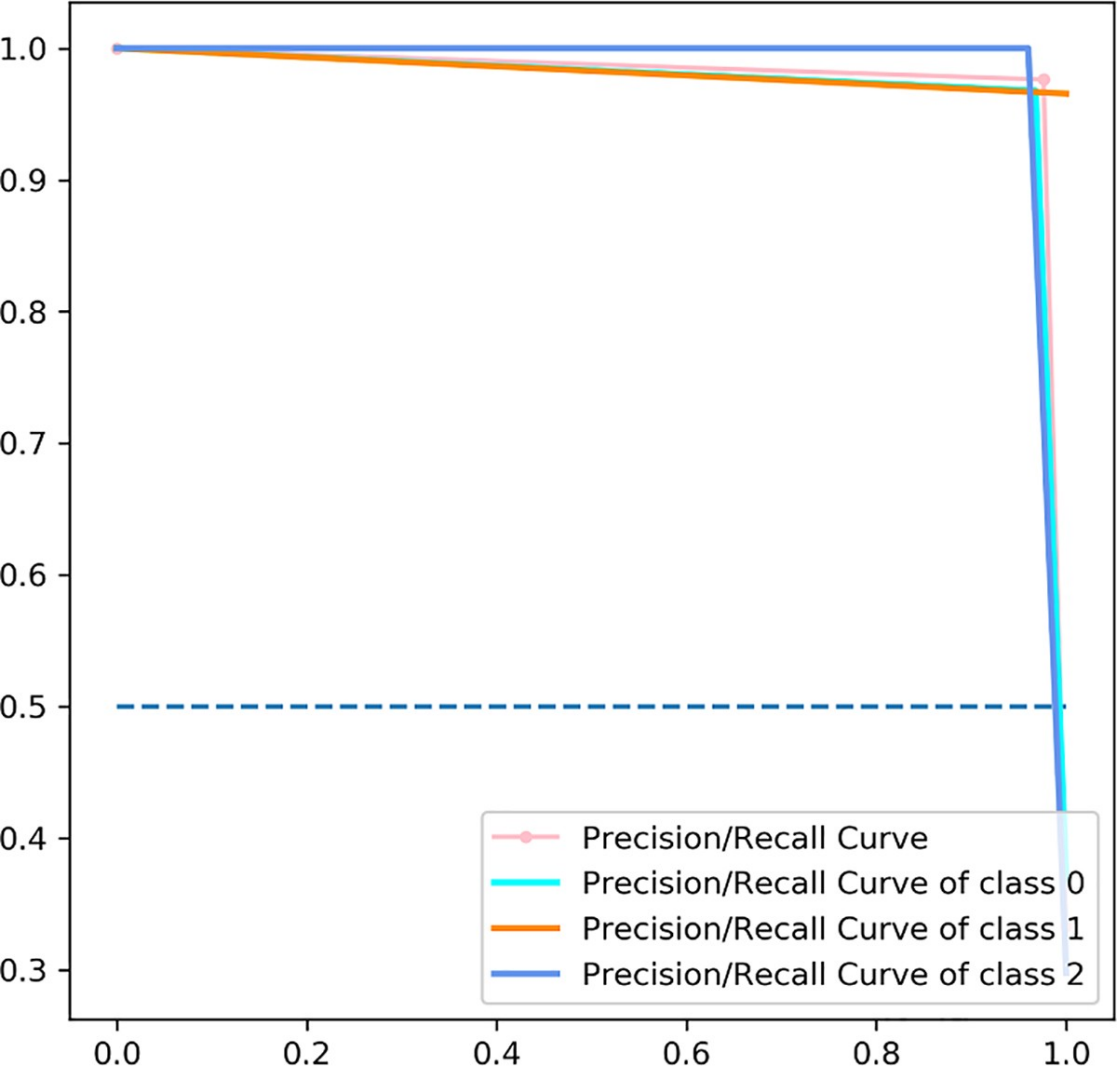
The precision/recall curves computed for CNN-LSTM method.

The ROC curves of the CNN-LSTM method are illustrated in Fig 7. The rate of false positive is near to zero while the rate of true positive is between 0.9 and 1. The precision-recall curves of the CNN-LSTM model can be observed in Fig 8. The precision-recall curve of our model shows the trade-off between precision and recall for different threshold. The high area under the curve represents both high recall and high precision, so we obtained a high precision meaning a low false positive rate, and high recall meaning a low false negative rate.

In the current pilot study, we propose a novel diagnosis classification method CNN-LSTM to characterize the focal pancreatic masses during the EUS procedure: the mixture between the CNN in order to better utilize spatial and configuration information of 2D images and the LSTM for the analysis of the contrast harmonic images to take into consideration the dependency relationships between the successive frames and extract sequential dynamic information which improved the accuracy of the overall result.

Other machine learning methods were developed before to assist endoscopists in the EUS evaluation of pancreatic lesions with similar results as our method. In a study by Zhu et al. [18], the support vector machine (SVM) predictive model was used to classify the EUS images for the differential diagnosis of PDAC and CP. The reported average accuracy, sensitivity, specificity, were 94.2%, 96.25%, 93.38, 92.21% and 96.68%, respectively. Das et al. [19] developed a neural network that identifies areas of pancreatic adenocarcinoma (PC) on EUS images. The trained ANN model based on eleven parameters extracted from EUS images was very accurate in classifying PDAC, with an AUC of 0.93. In a study by Ozkan et al. [20], an ANN model was proposed to classify malignant and non-malignant EUS images from patients with the age as under 40, between 40 and 60, and over 60. The obtained results were: accuracy: 92%, 88.5%, and 91.7%, respectively; sensitivity: 87.5%, 85.7%, and 93.3%, respectively; and specificity: 94.1%, 91.7%, and 88.9%, respectively. When all the age groups were used together, the following values were obtained: accuracy: 87.5%, sensitivity: 83.3%, and specificity: 93.3%. According to their results, better diagnostic performances were obtained when age ranges were separately examined.

The important improvements of deep learning over other machine learning techniques had a big impact in medical image diagnosis. Kuwahara et al. [21] evaluated the diagnosis of malignancy in intraductal papillary mucinous neoplasms of the pancreas using deep learning methods, with promising results. The AUC was 0.98, while the sensitivity, specificity, and accuracy were 95.7%, 92.6%, and 94.0%, respectively. Kurita et al. [22] investigated the diagnostic ability of carcinoembryonic antigen (CEA), cytology, and artificial intelligence (AI) by deep learning in differentiating malignant from benign cystic lesions. AUC for the diagnostic ability of malignant cystic lesions were 0.719 for CEA, 0.739 for cytology and 0.966 for AI. Accordingly, AI may improve the diagnostic ability in differentiating malignant from benign pancreatic cystic lesions.

In our study we used CNNs to extract the visual features of focal pancreatic masses because they can be adapted to their intrinsic structure, while the recurrent neural networks (RNNs), particularly LSTM was used to exploit the temporal information contained in EUS temporal images. The combination of CNNs and RNNs generated a model with an overall high accuracy of 98.26%. Although this represents an acceptable accuracy rate in the field, the algorithm will be further tested and improved on a larger patient database during future studies to address the current limited sample size and the lack of an independent test cohort.

## Conclusions

In the current pilot study, we have used an endoscopic ultrasound imaging data set to train the CNN-LSTM algorithm to generate the automatic, real-time diagnosis. A CNN model was developed in order to differentiate the visual features of chronic pseudotumoral pancreatitis, neuroendocrine tumor and ductal adenocarcinoma images. Furthermore, a LSTM network model was developed to capture the dynamics of the physical features over time. As a clinical decision supporting system, deep learning models could improve the differential diagnostic ability of pancreatic masses. Based on these promising preliminary results and further testing on a larger dataset, our method could become an important tool for the computer-aided diagnosis of focal pancreatic masses.

## Supporting information

S1 Fig Detailed architecture of CNN-LSTM model. (TIF)

S1 Table Parameters of the proposed CNN-LSTM architecture. (DOCX)

S1 File Supporting information file. (DOCX)
