# Supplementary figures and images for: Real-time computer-aided diagnosis of focal pancreatic masses from endoscopic ultrasound imaging based on a hybrid convolutional and long short-term memory neural network model
Source: PLoS One. 2021 Jun 28;16(6):e0251701. doi: 10.1371/journal.pone.0251701 (PMC8238220; doi:10.1371/journal.pone.0251701)

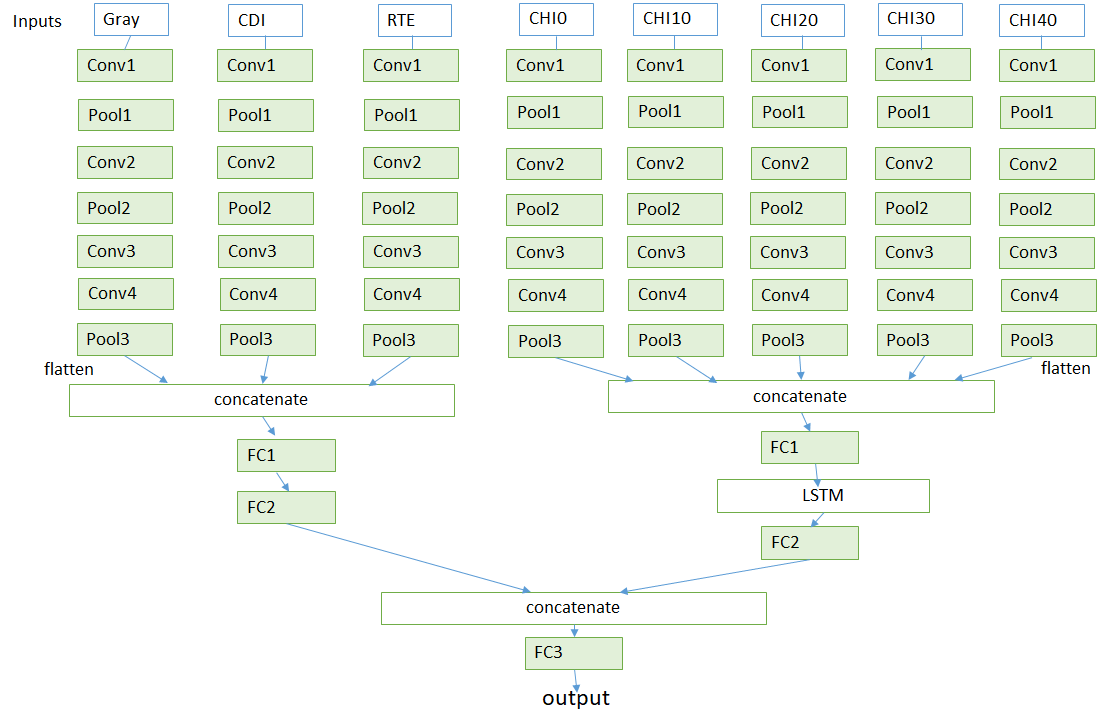

Supplement: S1 Fig — (TIF) [file pone.0251701.s001.tif]
