# Supplementary material for: Real-time computer-aided diagnosis of focal pancreatic masses from endoscopic ultrasound imaging based on a hybrid convolutional and long short-term memory neural network model
Source: PLoS One. 2021 Jun 28;16(6):e0251701. doi: 10.1371/journal.pone.0251701 (PMC8238220; doi:10.1371/journal.pone.0251701)
Supplement: S1 Table — (DOCX) [file pone.0251701.s002.docx]

S1 Table. Parameters of the proposed CNN-LSTM architecture

| **Inputs** | **Layers/**  **Parameters** | **Conv1** | **Pool1** | **Conv2** | **Pool2** | **Conv3** | **Conv4** | **Pool3** | **FC1** | **LSTM** | **FC2** | **FC3** |
| --- | --- | --- | --- | --- | --- | --- | --- | --- | --- | --- | --- | --- |
| CDI, GRAY  RTE | Kernel | 3*3 | 3*3 | 3*3 | 2*2 | 3*3 | 3*3 | 2*2 | - | - | - | - |
|  | Channel | 32 | 32 | 32 | 32 | 64 | 128 | 128 | 256 | - | 128 | 3 |
| CHI 0  CHI10  CHI20  CHI30  CHI40 | Kernel | 3*3 | 3*3 | 3*3 | 2*2 | 3*3 | 3*3 | 2*2 | - | - | - | - |
|  | Channel | 32 | 32 | 32 | 32 | 64 | 128 | 128 | 128 | 100 | 64 | 3 |

Conv: convolutional layer; Pool: pooling layer; FC: fully connected layer; LSTM: long short- term memory.
