# Supplementary material for: Real-time computer-aided diagnosis of focal pancreatic masses from endoscopic ultrasound imaging based on a hybrid convolutional and long short-term memory neural network model
Source: PLoS One. 2021 Jun 28;16(6):e0251701. doi: 10.1371/journal.pone.0251701 (PMC8238220; doi:10.1371/journal.pone.0251701)

S1 File

# Multiple input CNN- LSTM model architecture and parameters

The first CNN model has three inputs, one for each GRAY, CDI and RTE imaging modalities. The spatial features of images are extracted using the following CNN architecture with 3 inputs: 4 convolution layers with a feature map of size (3 x 3), 3 max-pooling layers with a pooling window of size (2 x 2) and a flatten layer. The vectors of images features from each modality are concatenated through a merge layer, which is followed by two fully connected layers.

The second CNN-LSTM model is developed for the CHI imaging modality. The CHI modality produces a sequence of 5 images, so we took into consideration the temporal changes to make predictions. We combined CNN with LSTM to determine the time dependencies in images. The arterial and venous images are combined as a single image dataset into the CNN.

The spatial features of input images taken at 0s, 10s, 20s, 30s and 40s (arterial and venous phases) are extracted using a CNN architecture with 5 inputs: 4 convolution layers with a feature map of size (3 x 3), 3 max-pooling layers with a pooling window of size (2 x 2) and a flatten layer.

The feature vectors from each imaging modalities (CHI 0s, CHI 10s, CHI 20s, CHI 30s, CHI 40 s) are concatenated through a merge layer which is followed by a fully connected layer. The output of CNN is passed to the LSTM layer with 100 inputs, which is followed by a fully connected layer.

The features vectors of the two models are concatenated through a merge layer. The last layer is a fully connected layer with three outputs, corresponding to the three diagnosis classes. The parameters of the method are presented in S1 Table.

S1 Table. Parameters of the proposed CNN-LSTM architecture

| **Inputs** | **Layers/**  **Parameters** | **Conv1** | **Pool1** | **Conv2** | **Pool2** | **Conv3** | **Conv4** | **Pool3** | **FC1** | **LSTM** | **FC2** | **FC3** |
| --- | --- | --- | --- | --- | --- | --- | --- | --- | --- | --- | --- | --- |
| CDI, GRAY  RTE | Kernel | 3*3 | 3*3 | 3*3 | 2*2 | 3*3 | 3*3 | 2*2 | - | - | - | - |
|  | Channel | 32 | 32 | 32 | 32 | 64 | 128 | 128 | 256 | - | 128 | 3 |
| CHI 0  CHI10  CHI20  CHI30  CHI40 | Kernel | 3*3 | 3*3 | 3*3 | 2*2 | 3*3 | 3*3 | 2*2 | - | - | - | - |
|  | Channel | 32 | 32 | 32 | 32 | 64 | 128 | 128 | 128 | 100 | 64 | 3 |

Conv: convolutional layer; Pool: pooling layer; FC: fully connected layer; LSTM: long short- term memory.

The detailed architecture of the proposed method is presented in S1 Fig.

S1 Fig. Detailed architecture of multiple input CNN-LSTM model.


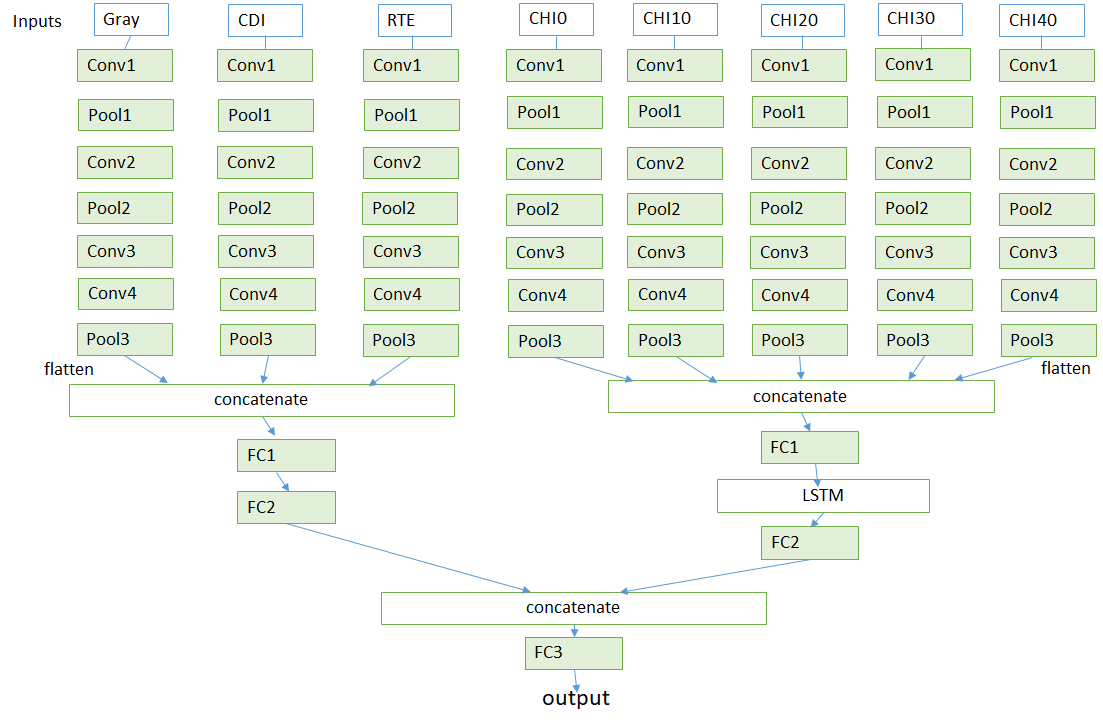

Supplement: S1 File — (DOCX) [file pone.0251701.s003.docx]
